# Non-Invasive Surfactant Administration in Preterm Infants

**DOI:** 10.3390/children13010150

**Published:** 2026-01-21

**Authors:** Faten Budajaja, Nadine Lahage, Ivan L. Hand

**Affiliations:** New York City Health & Hospitals/Kings County, SUNY Downstate Health Sciences University, Brooklyn, NY 11203, USA; faten.budajaja@downstate.edu (F.B.); nadine.lahage@nychhc.org (N.L.)

**Keywords:** respiratory distress syndrome, RDS, BPD, surfactant, InSurE, LMA, SALSA, oropharyngeal, nebulization, preterm

## Abstract

Background: Although surfactant replacement therapy has been a cornerstone of respiratory distress syndrome (RDS) management for decades, traditional delivery via endotracheal intubation and mechanical ventilation is associated with procedure-related complications and increased risk of bronchopulmonary dysplasia (BPD). These concerns have driven the development of less invasive surfactant administration strategies. Objective: This review aims to summarize and evaluate the current literature on less invasive surfactant delivery techniques used in preterm infants with RDS, with a focus on their feasibility, efficacy, and short- and long-term neonatal outcomes. Methods: We reviewed the available literature evaluating less invasive surfactant administration methods, including InSurE, Less Invasive Surfactant Therapy/Minimally Invasive Surfactant Therapy (LISA/MIST), surfactant administration via laryngeal mask airway (SALSA/LMA), pharyngeal administration, and nebulized surfactant. We compared major outcomes, namely the need for mechanical ventilation, incidence of BPD, procedural complications and long-term neurodevelopmental outcomes. Results: Non-invasive surfactant administration techniques have been associated with reduced exposure to mechanical ventilation and lower rates of BPD compared with conventional approaches. Studies on LISA/MIST demonstrate the most consistent evidence in reducing the need for mechanical ventilation and BPD, while other techniques such as LMA-assisted delivery and nebulization show promise but remain limited by device constraints, gestational age applicability, and heterogeneous study designs. Long-term neurodevelopmental outcome data remain sparse across all techniques. Conclusions: Non-invasive surfactant administration represents an important advancement in the management of RDS. While several techniques offer potential advantages over traditional intubation-based delivery, further high-quality studies are required to optimize patient selection, standardize techniques, develop safe and effective delivery devices, and evaluate long-term neurodevelopmental outcomes.

## 1. Introduction

Respiratory distress syndrome (RDS) is the most common respiratory disorder of preterm infants, with an incidence as high as 93% in those born below 28 weeks of gestation [1]. Surfactant replacement became a well-established, effective, and safe therapy by the early 1990s [2]. In preterm infants with respiratory distress, surfactant administration has shown improvements in survival rates and a decreased risk of chronic lung disease, as well as a reduced incidence of pneumothorax and pulmonary interstitial emphysema [3]. Surfactant acts by reducing alveolar surface tension, leading to a rapid improvement in lung volume, functional residual capacity, and compliance [4]. Surfactant replacement therapy may also have potential benefits as an adjunct treatment in conditions beyond RDS, such as meconium aspiration syndrome, neonatal pneumonia, and pulmonary hemorrhage, although current evidence remains limited [5].

There are multiple types of surfactant available, both synthetic or “natural” from porcine or bovine sources. Traditionally, surfactant was given through endotracheal intubation and mechanical ventilation. There are multiple risks associated with this procedure including desaturation, bradycardia, apnea, and hypertension and more severe risks such as perforation, infection, and pneumothorax [6]. In addition, prolonged use of an endotracheal tube is associated with an increased risk of developing bronchopulmonary dysplasia [7]. Since positive pressure ventilation itself has been recognized as a contributor to lung injury, less invasive methods of surfactant administration have been pursued.

Due to the aforementioned variety of intubation- and ventilation-associated complications, various less invasive techniques for administering surfactant have been used and continue to be explored. These methods include InSurE (Intubation, Surfactant, Extubation), to less invasive methods like LISA/MIST (Less Invasive Surfactant Therapy/Minimally Invasive Surfactant Therapy) using a thin catheter for administration, SALSA (Surfactant Administration through Laryngeal or Supraglottic Airways), oropharyngeal administration, and nebulization. All of these methods of non-invasive surfactant administration seek to deliver surfactant over a short time period while avoiding the risk of endotracheal intubation and mechanical ventilation [8]. Delivery of surfactant via non-invasive means also allows for preservation of glottic function and allows the surfactant to disperse by spontaneous breathing [9]. Despite these multiple emerging techniques, there remains significant variability in practice and limited high-quality comparative trials. These gaps highlight the need to synthesize current evidence on non-invasive strategies.

In this article, we review the different methods of non-invasive surfactant administration and discuss their efficacy on short- and long-term neonatal outcomes.

## 2. Methods

A narrative review of the literature was performed using PubMed and Google Scholar to identify randomized controlled trials, systematic reviews, and meta-analysis evaluating non-invasive surfactant administration in preterm neonates. Emphasis was placed on recent meta-analysis and large randomized controlled trials with significant clinical impact. Selected studies were reviewed and synthesized qualitatively to summarize current evidence and clinical implications.

## 3. Results: Non-Invasive Surfactant Administration

### 3.1. INSURE (Intubation, Surfactant, Extubation)

The InSurE (Intubate, Surfactant, Extubate) technique was first introduced by Verder et al. in 1994 [10]. In this study surfactant was administered via an endotracheal tube and patients were extubated after two to five minutes of manual ventilation, once adequate respiration was achieved. The InSurE technique involves brief endotracheal intubation to deliver a bolus of surfactant directly into the trachea, followed by short-term positive-pressure ventilation to facilitate surfactant distribution and prompt extubation without continuation of invasive mechanical ventilation [8]. This approach is typically applied in preterm infants with RDS who are receiving nCPAP with oxygen requirements (generally FiO_2_ ≥ 0.30–0.40), are spontaneously breathing and hemodynamically stable, and do not exhibit significant apnea or respiratory failure necessitating ongoing ventilatory support [8,11]. Successful InSurE requires skilled personal experience in neonatal endotracheal intubation, rapid surfactant administration, and immediate extubation [12]. Premedication for intubation may be used in accordance with institutional protocols, with consideration given to analgesia or sedation strategies that preserve spontaneous respiration [8,13]. Essential equipment includes appropriately sized endotracheal tubes according to gestational age and weight, a laryngoscope with an appropriate blade, and end-tidal CO_2_ detection to confirm correct endotracheal tube placement. This strategy was found to minimize the duration of mechanical ventilation and reduce the subsequent need for intubation [10]. A follow-up study of 60 infants born at less than 30 weeks’ gestation by Verder [14] demonstrated similarly favorable outcomes, showing a significant reduction in the need for mechanical ventilation among preterm infants treated with early selective surfactant using the InSurE technique. In support of these findings, a subsequent study by Dani et al. in 2004 reported that resuming NCPAP (nasal continuous positive airway pressure) immediately after surfactant in infants with RDS was safe and reduced the need for mechanical ventilation, duration of respiratory support, surfactant use, and NICU stay, lowering the overall cost of care [15]. A subsequent Cochrane review of the InSurE technique, including six randomized controlled trials, showed a reduced need for mechanical ventilation, a decreased incidence of bronchopulmonary dysplasia, and fewer air leak syndromes [16]. Although these studies revealed significant improvement in outcomes using the InSurE technique, this procedure is still considered invasive and requires advanced skills as well as a period of mechanical ventilation.

### 3.2. LISA/MIST (Less Invasive Surfactant Therapy/Minimally Invasive Surfactant Therapy)

The first published mention of the LISA technique was from Verder who first used a small-diameter gastric tube to instill surfactant while the infant was spontaneously breathing in 1992 [17]. Kribs and colleagues reintroduced LISA 15 years later by using laryngoscopes and thin feeding tubes in infants on CPAP [18]. In this technique, use of an endotracheal tube and manual ventilation is avoided. LISA/MIST are bedside techniques that enable surfactant delivery to spontaneously breathing preterm infants receiving non-invasive respiratory support, most commonly nCPAP. Both techniques involve brief laryngoscopy and placement of a thin catheter through the vocal cords, allowing surfactant administration while avoiding intubation and mechanical ventilation. Successful implementation requires performance by a skilled provider experienced in neonatal airway management to minimize procedural stress and complications. Premedication practices vary across centers; when used, medications are selected to reduce discomfort while preserving spontaneous breathing, as excessive sedation may increase the risk of respiratory depression and procedural failure [18,19]. This method of less invasive surfactant administration offers observed advantages over standard surfactant therapy, including avoidance of mechanical ventilation and associated lung injury, a reduced need for sedation-and intubation-related complications, preservation of physiological spontaneous breathing, and potentially a lower rate of bronchopulmonary dysplasia [20]. In the Optimist trial by Dargaville et al. in 2021, a more rigid adult vascular catheter was used, employing the Hobart method, to avoid using Magill forceps [21].

Multiple RCTs have been performed to compare LISA/MIST to InSurE or standard surfactant therapy in the preterm newborn population. A large parallel-group controlled trial conducted across 12 NICUs evaluated 220 preterm infants born between 26 and 28 weeks’ gestation with birth weights < 1500 g [22]. The study compared surfactant administration via 5Fr thin catheter with Magill forceps and laryngoscopes to standard surfactant administration. The study revealed a significant decrease in the need for mechanical ventilation in the intervention group (28% vs. 46%). Additionally, the total number of ventilation days was markedly lower in the intervention group, with 242 days compared to 599 days in the control group. In the 2015 NINSAPP trial by Kribs et al., 211 infants born at 23–26 weeks’ gestation were randomized to compare LISA to standard intubation and demonstrated a significant reduction in pneumothorax, intraventricular hemorrhage, and the need for mechanical ventilation [23]. In the landmark, multi-center OPTIMIST-A randomized controlled trial led by Dargaville, the effectiveness of minimally invasive surfactant therapy (MIST) in extremely preterm infants was evaluated. Conducted between 2011 and 2020 across 33 NICUs internationally, the trial enrolled 485 infants born between 25 and 28 + 6 weeks’ gestation, who were randomized to receive MIST or the SHAM procedure. The study demonstrated that MIST significantly reduced bronchopulmonary dysplasia among survivors, as well as several key morbidities, including the need for mechanical ventilation within 72 h and the incidence of pneumothorax. However, despite these respiratory benefits, the intervention did not reduce the composite outcome of death or BPD, as mortality rates were similar between groups. Overall, this large, rigorously conducted trial provides the strongest evidence to date that non-invasive surfactant delivery via MIST can mitigate important neonatal pulmonary complications [21].

Similarly, Katheria et al. in 2023 enrolled 180 infants at 24–29 weeks to compare LISA plus caffeine to NCPAP plus caffeine, showing a lower rate of bronchopulmonary dysplasia and a significant reduction in the need for intubation [24].

A recent meta-analysis by Veintemilla [25] in 2025 analyzed 17 studies of preterm infants < 37 weeks, testing the efficacy and safety of MIST compared to the INSURE technique in 1931 neonates. They concluded a significant reduction in the need for mechanical ventilation but without a significant difference in mortality rate between both groups. There have been various studies highlighting the clinical benefit of LISA/MIST in reducing ventilatory burden in preterm infants [26,27,28], as well as a decrease in hospital stay days [29,30]. There have also been concerns raised over the use of sedating medications in these studies. Using sedating premedication has been linked with delayed extubation due to respiratory suppression [31]. This is supported by the finding of Dekker, where low-dose sedation improved comfort during the MIST procedure but increased the need for transient non-invasive ventilation [32].

Hospital admission characteristics, psychomotor development, and respiratory and neurological outcomes were evaluated at 24 months in a single-center, retrospective study including 60 preterm infants receiving nCPAP within the first 3 days of life and requiring surfactant therapy. Infants were managed using either the InSurE technique or LISA. No significant differences were observed between the two groups with respect to psychomotor development or long-term respiratory and neurological outcomes [33].

Although LISA/MIST are considered less invasive methods of surfactant administration because they allow spontaneous breathing and have been shown to significantly reduce the need for mechanical ventilation, these techniques still require a high level of provider experience and technique. In addition, the catheters used remain off-label for this purpose. Further research is needed to refine these approaches and to compare their safety and effectiveness with other truly non-invasive surfactant delivery methods.

### 3.3. Nebulization/Aerosolized Surfactant

In 1964, Robillard and colleagues were the first to explore aerosolized delivery of synthetic surfactant—specifically nebulized dipalmitoyl-phosphatidylcholine—administered directly into the infant incubator [34]. Although the results were not significant, this method has pushed the field to explore more effective ways of surfactant administration. Several studies were performed subsequently. Cummings et al. conducted one of the largest trials evaluating surfactant using a modified Solaris nebulizer coupled with a pacifier-style interface [35]. The study enrolled 457 infants between 23 and 41 weeks’ gestation, including 401 preterm infants < 37 weeks, to assess whether nebulized surfactant could reduce the need for intubation. The intervention was associated with a significant reduction in intubation within 72 h of life (*p* = 0.0001), with the greatest benefit observed in infants with mild to moderate respiratory distress. Although the findings suggest that nebulized surfactant may be a feasible and effective non-invasive strategy to support spontaneous breathing and avoid mechanical ventilation, the trial had notable methodological limitations. These included an unblinded design and the absence of pre-specified, standardized criteria for intubation, which may have introduced bias in clinical decision-making. Despite these limitations, the study provides important evidence supporting the potential role of nebulized surfactant as a gentler alternative to traditional surfactant delivery approaches.

In 2021, Gaertner performed a meta-analysis including nine studies, finding no differences in neonatal morbidities and mortality in nebulized surfactant versus standard care groups. They did find a difference in the need for neonatal intubation in certain subgroups [36].

Nebulized or aerosolized surfactant represents a fully non-invasive approach to surfactant delivery in preterm infants with RDS who are breathing spontaneously on non-invasive respiratory support, such as nCPAP or nasal intermittent positive-pressure ventilation [36]. In clinical practice, surfactant is administrated using specialized nebulization devices, such as a vibrating mesh nebulizer, integrated into the non-invasive respiratory circuit [37,38]. This technique avoids laryngoscopy and airway instrumentation, thereby reducing procedural stress and eliminating the need for endotracheal intubation or catheter placement. Effective delivery depends on multiple technical factors, including nebulizer type, particle size, circuit configuration, driving pressure, airflow velocity, and synchronization with spontaneous breathing. Experimental data suggest that an optimal particle size of approximately 3–4 μm is required to enhance alveolar deposition while minimizing loss in the upper airway [39]. Current evidence indicates that nebulized surfactant is feasible and generally well-tolerated, with minimal acute adverse events. However, its clinical efficacy remains variable, likely due to limited and inconsistent lung deposition compared with intratracheal surfactant administration. Multiple nebulizer systems, including jet, ultrasonic, and vibrating membrane devices, have been investigated, but regulatory approval for surfactant nebulization has not yet been granted [40]. As a result, nebulized surfactant is primarily studied as an early or adjunctive strategy within clinical trials or specialized protocols, and further large, randomized controlled trials are needed before widespread clinical adoption.

Nebulized surfactant aims to deliver surfactant to the lungs in a fine mist that can be inhaled by infants, avoiding endotracheal intubation and mechanical ventilation [32]. The efficacy of aerosolized surfactant remains unresolved due to the considerable technical challenges associated with ensuring adequate lung deposition of nebulized surfactant in preterm infants. Multiple nebulizer systems have been studied, including jet and ultrasonic nebulizers and vibrating membrane nebulizers; however, FDA approval for surfactant nebulization has not yet been granted [33]. In addition to that, adequate alveolar delivery of aerosolized surfactant depends on droplet size, driving pressure, and airflow velocity. Some data show that the ideal particle size is about 3–4 μm [34]. These factors are important to optimize surfactant delivery to the lung and decrease its deposition in mouth and upper airway. Thus, although aerosolized surfactant is completely non-invasive, avoiding airway manipulation and sedation, and remains a promising technique, it has limitations due to limited lung deposition.

### 3.4. Oropharyngeal Delivery

Oropharyngeal surfactant delivery is a non-invasive technique in which surfactant is instilled into the oropharynx immediately after birth, prior to the initiation of effective spontaneous respiration, with the aim of facilitating pulmonary distribution during the infant’s first breaths [41]. This approach avoids laryngoscopy, endotracheal intubation, and positive-pressure ventilation, thereby minimizing airway manipulation and procedural stress. In clinical practice, a predetermined dose of surfactant is gently administered into the posterior oropharynx using a syringe during the immediate postnatal period, typically while the infant is maintained with non-invasive respiratory support such as nCPAP [35]. The oropharyngeal method is simpler and cheaper and causes less discomfort than either InSurE or LISA. In 2024, the POPART study, a randomized controlled trial conducted across nine European NICUs, included 252 infants born at 26–28 weeks’ gestation and evaluated prophylactic oropharyngeal surfactant. The trial found no significant difference in the primary outcome of mechanical ventilation within the first 120 h, and no improvement in secondary outcomes. Notably, there was an increased risk of pneumothorax in the intervention group [42]. Available evidence suggests that oropharyngeal surfactant administration is feasible and well-tolerated, with minimal reported adverse events. However, clinical efficacy remains inconsistent, likely due to variability in surfactant aspiration and lung deposition [43]. As a result, this method remains an investigational strategy and future research on oropharyngeal delivery must focus on optimizing the dose, timing, and subgroup populations in evaluating efficacy in extremely premature infants.

### 3.5. SALSA (Surfactant Administration Through Laryngeal or Supraglottic Airways)

The supraglottic device (SAD) history started in 1981 by Dr Brain, an anesthesiologist in London. SAD was used in the management of difficult airways by avoiding face masks and intubation in adult patients [44,45]. Neonatal resuscitation using a laryngeal mask was first described in 1994, allowing for successful resuscitation in 20 neonates [46]. In the 2005 American Heart Association (AHA) guidelines for CRP and Emergency Cardiovascular Care, the laryngeal mask airway was adopted as an acceptable advanced airway device to be used by trained providers as an alternative to the face mask and endotracheal intubation during resuscitation [47].

Surfactant delivery via a laryngeal mask airway (LMA) is an alternative non-invasive technique for select preterm infants with RDS, allowing surfactant instillation without endotracheal intubation and mechanical ventilation. An LMA is a supraglottic airway device that sits above the vocal cords and can be used as a conduit for surfactant administration in spontaneously breathing infants supported on non-invasive ventilation such as CPAP. Most clinical evidence supports use in preterm infants ≥ 28 weeks gestation and ≥1200 g with clinical and radiographic evidence of RDS who are managed initially on non-invasive support [48].

The first documented use of a laryngeal mask airway for surfactant administration was reported by Brimacome in 2004, describing successful delivery of surfactant to two preterm infants with respiratory distress syndrome [49]. This was followed by a larger study published by Trevisanuto and colleagues in 2005, who administered surfactant via LMA in eight preterm infants, demonstrating feasibility and clinical improvement without the need for endotracheal intubation. These early reports laid the foundation for subsequent studies exploring LMA as a non-invasive alternative for surfactant delivery in infants with RDS [50].

In 2013, Attridge et al. conducted a randomized controlled pilot trial involving 26 preterm infants with birth weights > 1200 g, comparing surfactant administration via LMA to continued standard nasal CPAP without surfactant. The study demonstrated that infants in the LMA group had a significant reduction in supplemental oxygen requirements at both 1 h and 12 h after randomization [51]. A larger, prospective, multi-center, randomized controlled trial compared LMA surfactant delivery versus standard continuous nasal CPAP without surfactant, enrolling 103 preterm infants aged 28–35 weeks, with birth weights > 1250 g. The study demonstrated a significant decrease in the rate of intubation and mechanical ventilation in the LMA group compared with the control group, with no serious adverse events [52].

Subsequently, there have been multiple randomized controlled trials comparing laryngeal mask surfactant administration with endotracheal tube administration. Barbosa et al. included 48 preterm infants with gestational ages 28–35 weeks and birth weights > 1000 g in a trial comparing surfactant administration via LMA versus ETT with immediate extubation. The trial demonstrated a more rapid reduction in oxygen needs in infants treated with LMA, achieving FiO_2_ < 0.30 within 3 h of surfactant administration. Additionally, they reported gastric residual volumes > 1.5 mL after LMA surfactant delivery [53].

Kubicka conducted an observational cohort study involving 120 infants, aged 28–41 weeks, with birth weights > 1250 g, who received surfactant via LMA as a first-line therapy. The study reported a 70% avoidance of intubation and invasive ventilation, along with a reduced rate of pneumothorax [54].

In the most recent Cochrane review of eight trials published up to December 2022, surfactant administration via LMA was compared with InSurE, endotracheal delivery, and no surfactant. Rescue S-LMA appeared to have little to no impact on death or BPD at 36 weeks but may reduce the need for mechanical ventilation, particular in studies where the comparison groups received sedation or analgesia. No significant differences were observed in other neonatal morbidities or mortality, and long-term outcomes remain largely unknown. For infants <32 weeks or <1500 g, the available evidence is insufficient to determine the effectiveness of surfactant administration via LMA [55], and long-term outcomes remain largely unreported in the existing literature [55]. The SALSA—LMA approach is simple and feasible, requiring minimal technical skills. However, further studies, particular in infants of younger gestational ages, are needed to establish its safety and effectiveness for long-term as well as short-term outcomes.

## 4. Discussion

With advances in perinatal and neonatal care, the survival of extremely preterm infants has increased, particularly among those born at the limits of viability (approximately 22 weeks’ gestation) [56]. However, as gestational age decreases, the risk for chronic lung disease rises substantially, making surfactant therapy an essential component of respiratory management in this vulnerable population [57]. Consequently, significant efforts have focused on developing surfactant delivery strategies that minimize lung injury and procedure-related adverse events. LISA/MIST has been increasingly adopted because it allows delivery of surfactant without interrupting spontaneous breathing and may decrease the rate of mechanical ventilation and some morbidities compared with more invasive methods [58]. Despite these advances, important challenges persist across all currently available techniques. Airway manipulation using a laryngoscope remains a key limitation of both the InSurE and LISA/MIST approaches, potentially triggering physiological instability and stress responses [52]. In addition, the need for premedication, including sedation and analgesia, raises concerns regarding respiratory depression, hemodynamic effects, and potential effects on neurodevelopment [58]. Technical barriers also remain, including the identification of optimal devices and aerosol particle sizes to achieve effective and uniform pulmonary surfactant deposition. Current supraglottic devices are limited by size and weight criteria, restricting their use in the most immature infants [59,60]. Recent clearance, however, by the U.S Food and Drug Administration of a novel supraglottic airway device for neonates weighing less than 1000 g may broaden therapeutic options and reduce reliance on endotracheal intubation in extremely-low-birth-weight infants [60]. As previously discussed, InSurE and LISA/MIST techniques require a high level of provider expertise, which may limit both their feasibility and widespread implementation, particularly in resource-limited settings [61]. Taken together, these limitations highlight that less invasive surfactant delivery methods represent meaningful progress but no single approach has yet emerged as universally optimal (Table 1).

Future research should prioritize well-designed, adequately powered randomized controlled trials comparing surfactant delivery techniques across gestational ages, with particular emphasis on extremely preterm infants. In addition to short-term respiratory outcomes, these studies should incorporate standardized assessments of long-term respiratory health, neurodevelopmental outcomes, and quality of life extending into early childhood and beyond. Such data are essential to determine the true safety, efficacy, and generalizability of less invasive surfactant administration strategies that optimize both survival and long-term outcomes in this high-risk population.

## 5. Conclusions

Surfactant administration has evolved considerably over the past 30 years. Although these newer, less invasive methods of delivery demonstrate certain advantages compared with standard intubation or INSURE, further research is essential, particularly in the most immature preterm infants to establish and validate the most effective clinical practice

## Figures and Tables

**Table 1:** Summary of non-invasive surfactant administration methods.

Method	Route of Administration/Typical Gestational Age	Invasiveness/Required Skills	Ventilation/Spontaneous Breathing	Premedication	Notes
InSurE	ETT/no strict cutoff gestational age	Invasive/advanced skills	Brief MV	Usually required	Widely studied. Decreases MV duration.
Nebulizing surfactant (aerosolized)	Inhalation via nebulizer/explored in infants 24–36 weeks	Non-invasive/no skills	Spontaneous breathing	Not required	Avoids airway manipulation.
Oropharyngeal surfactant	Instilled into oropharynx/in trial setting ≤ 29 weeks	Minimally invasive/minimal skills	Spontaneous breathing	Not required	Simple. Did not improve primary outcome.
LISA/MIST	Thin catheter into trachea/studied in ≥25 weeks	Less invasive/advanced skills	Spontaneous breathing	Depending on institutional protocol	Less need for MV. Decreases BPD.
LMA	Supraglottic airway/studied in ≥27 weeks, ≥800 g	Minimally invasive/less skills	Manual breaths	Usually not required	Feasible. Limited data in extremely preterm infants.

## Data Availability

No new data were created or analyzed in this study.

## References

[1] Zysman-Colman Z., Tremblay G.M., Bandeali S., Landry J.S. (2013). Bronchopulmonary dysplasia—Trends over three decades. Paediatr. Child Health.

[2] Engle W.A., American Academy of Pediatrics Committee on Fetus and Newborn (2008). Surfactant-replacement therapy for respiratory distress in the preterm and term neonate. Pediatrics.

[3] Suresh G.K., Soll R.F. (2003). Exogenous surfactant therapy in newborn infants. Ann. Acad. Med. Singap..

[4] Halliday H.L. (2008). Surfactants: Past, present and future. J. Perinatol..

[5] Finer N.N. (2004). Surfactant use for neonatal lung injury: Beyond respiratory distress syndrome. Paediatr. Respir. Rev..

[6] Pamukcu U., Dal A., Altuntas N., Cınar C., Altunkaynak B., Peker I. (2020). Knowledge, behavior, and awareness of neonatologists and anesthesiologists about oral complications of intubation and protection methods. Int. Dent. J..

[7] Dumpa V., Northrup V., Bhandari V. (2011). Type and timing of ventilation in the first postnatal week is associated with bronchopulmonary dysplasia/death. Am. J. Perinatol..

[8] Sweet D.G., Carnielli V.P., Greisen G., Hallman M., Klebermass-Schrehof K., Ozek E., Te Pas A., Plavka R., Roehr C.C., Saugstad O.D. (2023). European Consensus Guidelines on the Management of Respiratory Distress Syndrome: 2022 Update. Neonatology.

[9] Härtel C., Kribs A., Göpel W., Dargaville P., Herting E. (2024). Less Invasive Surfactant Administration for Preterm Infants—State of the Art. Neonatology.

[10] Verder H., Robertson B., Greisen G., Ebbesen F., Albertsen P., Lundstrøm K., Jacobsen T. (1994). Surfactant therapy and nasal continuous positive airway pressure for newborns with respiratory distress syndrome. Danish-Swedish Multicenter Study Group. N. Engl. J. Med..

[11] Abdallah Y., Mkony M., Noorani M., Moshiro R., Bakari M., Manji K. (2023). CPAP failure in the management of preterm neonates with respiratory distress syndrome where surfactant is scarce. A prospective observational study. BMC Pediatr..

[12] Liu S., Wang Y., Zhu X., Chen F., Shi Y. (2024). Comparative efficacy and safety of pulmonary surfactant delivery strategies in neonatal RDS: A network meta-analysis. BMC Pulm. Med..

[13] Ayed M., Shah V.S., Taddio A. (2017). Premedication for non-urgent endotracheal intubation for preventing pain in neonates. Cochrane Database Syst. Rev..

[14] Verder H., Albertsen P., Ebbesen F., Greisen G., Robertson B., Bertelsen A., Agertoft L., te Djernes B., Nathan E., Reinholdt J. (1999). Nasal Continuous Positive Airway Pressure and Early Surfactant Therapy for Respiratory Distress Syndrome in Newborns of Less Than 30 Weeks’ Gestation. Pediatrics.

[15] Dani C., Bertini G., Pezzati M., Cecchi A., Caviglioli C., Rubaltelli F.F. (2004). Early extubation and nasal continuous positive airway pressure after surfactant treatment for respiratory distress syndrome among preterm infants <30 weeks’ gestation. Pediatrics.

[16] Stevens T.P., Harrington E.W., Blennow M., Soll R.F. (2007). Early surfactant administration with brief ventilation vs. selective surfactant and continued mechanical ventilation for preterm infants with or at risk for respiratory distress syndrome. Cochrane Database Syst. Rev..

[17] Verder H., Agertoft L., Albertsen P., Christensen N.C., Curstedt T., Ebbesen F., Greisen G., Hobolth N., Holm V., Jacobsen T. (1992). Surfaktantbehandling af nyfødte med respiratorisk distress-syndrom primaert behandlet med nasalt kontinuerligt positivt luftvejstryk. En pilotundersøgelse [Surfactant treatment of newborn infants with respiratory distress syndrome primarily treated with nasal continuous positive air pressure. A pilot study]. Ugeskr Laeger..

[18] Kribs A., Pillekamp F., Hünseler C., Vierzig A., Roth B. (2007). Early administration of surfactant in spontaneous breathing with nCPAP: Feasibility and outcome in extremely premature infants (postmenstrual age </=27 weeks). Pediatr. Anesth..

[19] Shim G.H. (2017). Update of minimally invasive surfactant therapy. Korean J. Pediatr..

[20] Lau C.S.M., Chamberlain R.S., Sun S. (2017). Less Invasive Surfactant Administration Reduces the Need for Mechanical Ventilation in Preterm Infants: A Meta-Analysis: A Meta-Analysis. Glob. Pediatr. Health.

[21] Dargaville P.A., Kamlin C.O.F., Orsini F., Wang X., De Paoli A.G., Kutman H.G.K., Cetinkaya M., Kornhauser-Cerar L., Derrick M., Özkan H. (2021). Effect of Minimally Invasive Surfactant Therapy vs Sham Treatment on Death or Bronchopulmonary Dysplasia in Preterm Infants With Respiratory Distress Syndrome: The OPTIMIST-ARandomized Clinical Trial. JAMA.

[22] Göpel W., Kribs A., Ziegler A., Laux R., Hoehn T., Wieg C., Siegel J., Avenarius S., von der Wense A., Vochem M. (2011). Avoidance of mechanical ventilation by surfactant treatment of spontaneously breathing preterm infants (AMV): An open-label, randomised, controlled trial. Lancet.

[23] Kribs A., Roll C., Göpel W., Wieg C., Groneck P., Laux R., Teig N., Hoehn T., Böhm W., Welzing L. (2015). Nonintubated Surfactant Application vs Conventional Therapy in Extremely Preterm Infants: A Randomized Clinical Trial. JAMA Pediatr..

[24] Katheria A., Ines F., Banerji A., Hopper A., Uy C., Chundu A., Coughlin K., Hutson S., Morales A., Sauberan J. (2023). Caffeine and Less Invasive Surfactant Administration for Respiratory Distress Syndrome of the Newborn. NEJM Évid..

[25] Veintemilla-Burgos F., Minchalo-Ochoa G., Balda S., Diaz-Djevoich I., Kronfle R., Panchana-Lascano M., Leone-Berry T. (2025). Tiny Lungs, Big Decisions: A Meta-Analysis Comparing Minimally Invasive Surfactant Therapy Versus Intubation-Surfactant-Extubation in Preterm Neonates with Respiratory Distress Syndrome. Int. J. Pediatr..

[26] Kanmaz H.G., Erdeve O., Canpolat F.E., Mutlu B., Dilmen U. (2013). Surfactant administration via thin catheter during spontaneous breathing: Randomized controlled trial. Pediatrics.

[27] Jomeh R.G., Boskabadi H., Maamouri G., Zakerihamidi M. (2019). Comparative Study of the Effect of the Administration of Surfactant through a Thin Endotracheal Catheter into Trachea During Spontaneous Breathing with Intubation (Intubation-Surfactant-Extubation Method). J. Clin. Neonatol..

[28] Olivier F., Nadeau S., Bélanger S., Julien A.S., Massé E., Ali N., Caouette G., Piedboeuf B. (2017). Efficacy of minimally invasive surfactant therapy in moderate and late preterm infants: A multicentre randomized control trial. Paediatr. Child Health.

[29] Gupta B.K., Saha A.K., Mukherjee S., Saha B. (2020). Minimally invasive surfactant therapy versus InSurE in preterm neonates of 28 to 34 weeks with respiratory distress syndrome on non-invasive positive pressure ventilation-a randomized controlled trial. Eur. J. Pediatr..

[30] Jena S.R., Bains H.S., Pandita A., Verma A., Gupta V., Kallem V.R., Abdullah M., Kawdiya A. (2019). On Behalf of Sure Group. Surfactant therapy in premature babies: SurE or InSurE. Pediatr. Pulmonol..

[31] Abdel-Latif M.E., Davis P.G., Wheeler K.I., De Paoli A.G., Dargaville P.A. (2021). Surfactant therapy via thin catheter in preterm infants with or at risk of respiratory distress syndrome. Cochrane Database Syst. Rev..

[32] Dekker J., Lopriore E., van Zanten H.A., Tan R.N.G.B., Hooper S.B., Te Pas A.B. (2019). Sedation during minimal invasive surfactant therapy: A randomised controlled trial. Arch. Dis. Child. Fetal Neonatal Ed..

[33] Márquez Isidro E., Sánchez Luna M., Ramos-Navarro C. (2019). Long-term outcomes of preterm infants treated with less invasive surfactant technique (LISA). J. Matern. Neonatal Med..

[34] Robillard E., Alarie Y., Dagenais-Perusse P., Baril E., Guilbeault A. (1964). Microaerosol Administration of Synthetic Beta-gamma-dipalmitoyl L-aplha lecithin in the Respiratory Distress Syndrome: A Prelimiinary report. Can. Med. Assoc. J..

[35] Cummings J.J., Gerday E., Minton S., Katheria A., Albert G., Flores-Torres J., Famuyide M., Lampland A., Guthrie S., Kuehn D. (2020). Aerosolized Calfactant for Newborns with Respiratory Distress: A Randomized Trial. Pediatrics.

[36] Gaertner V.D., Thomann J., Bassler D., Rüegger C.M. (2021). Surfactant Nebulization to Prevent Intubation in Preterm Infants: A Systematic Review and Meta-analysis. Pediatrics.

[37] Minocchieri S., Berry C.A., Pillow J.J., CureNeb Study Team (2019). Nebulised surfactant to reduce severity of respiratory distress: A blinded, parallel, randomised controlled trial. Arch. Dis. Child. Fetal Neonatal Ed..

[38] Abdel-Latif M.E., Osborn D.A. (2012). Nebulised surfactant in preterm infants with or at risk of respiratory distress syndrome. Cochrane Database Syst. Rev..

[39] Sood B.G., Cortez J., Kolli M., Sharma A., Delaney-Black V., Chen X. (2019). Aerosolized surfactant in neonatal respiratory distress syndrome: Phase I study. Early Hum. Dev..

[40] Brasher M., Raffay T.M., Cunningham M.D., Abu Jawdeh E.G. (2021). Aerosolized Surfactant for Preterm Infants with Respiratory Distress Syndrome. Children.

[41] Dargaville P.A., Aiyappan A., De Paoli A.G., Kuschel C.A., Kamlin C.O., Carlin J.B., Davis P.G. (2013). Minimally-invasive surfactant therapy in preterm infants on continuous positive airway pressure. Arch. Dis. Child. Fetal Neonatal Ed..

[42] Murphy M.C., Galligan M., Molloy B., Hussain R., Doran P., O’Donnell C. (2020). Study protocol for the POPART study-Prophylactic Oropharyngeal surfactant for Preterm infants: A Randomised Trial. BMJ Open.

[43] Abdel-Latif M.E., Osborn D.A. (2011). Pharyngeal instillation of surfactant before the first breath for prevention of morbidity and mortality in preterm infants at risk of respiratory distress syndrome. Cochrane Database Syst. Rev..

[44] Brain A.I. (1983). The laryngeal mask—A new concept in airway management. Br. J. Anaesth..

[45] Hernandez M.R., Klock P.A., Ovassapian A. (2012). Evolution of the extraglottic airway: A review of its history, applications, and practical tips for success. Anesthesia Analg..

[46] Paterson S.J., Byrne P.J., Molesky M.G., Seal R.F., Finucane B.T. (1994). Neonatal resuscitation using the laryngeal mask airway. Anesthesiology.

[47] American Heart Association (2006). 2005 American Heart Association (AHA) Guidelines for Cardiopulmonary Resuscitation (CPR) and Emergency Cardiovascular Care (ECC) of Pediatric and Neonatal Patients: Pediatric Basic Life Support. Pediatrics.

[48] Guthrie S.O., Fort P., Roberts K.D. (2021). Surfactant Administration Through Laryngeal or Supraglottic Airways. NeoReviews.

[49] Brimacombe J., Gandini D., Keller C. (2004). The laryngeal mask airway for administration of surfactant in two neonates with respiratory distress syndrome. Pediatr. Anesth..

[50] Trevisanuto D., Grazzina N., Ferrarese P., Micaglio M., Verghese C., Zanardo V. (2005). Laryngeal mask airway used as a delivery conduit for the administration of surfactant to preterm infants with respiratory distress syndrome. Biol. Neonatol..

[51] Attridge J.T., Stewart C., Stukenborg G.J., Kattwinkel J. (2013). Administration of rescue surfactant by laryngeal mask airway: Lessons from a pilot trial. Am. J. Perinatol..

[52] Roberts K.D., Brown R., Lampland A.L., Leone T.A., Rudser K.D., Finer N.N., Rich W.D., Merritt T.A., Czynski A.J., Kessel J.M. (2018). Laryngeal Mask Airway for Surfactant Administration in Neonates: A Randomized, Controlled Trial. J. Pediatr..

[53] Barbosa R.F., Simões ESilva A.C., Silva Y.P. (2017). A randomized controlled trial of the laryngeal mask airway for surfactant administration in neonates. J. Pediatr..

[54] Kubicka Z., Zahr E., Feldman H.A., Rousseau T., Welgs T., Ditzel A., Perry D., Lacy M., O’Rourke C., Arzuaga B. (2025). Feasibility and safety of surfactant administration via laryngeal mask airway as first-line therapy for a select newborn population: Results of a standardized clinical protocol. J. Perinatol..

[55] Abdel-Latif M.E., Walker E., Osborn D.A. (2024). Laryngeal mask airway surfactant administration for prevention of morbidity and mortality in preterm infants with or at risk of respiratory distress syndrome. Cochrane Database Syst. Rev..

[56] Queliz T., Perez J.A., Corrigan M.J. (2021). A comparison of LISA versus InSurE: A single center experience. J. Neonatal-Perinatal. Med..

[57] Polin R.A., Carlo W.A., Papile L.-A., Carlo W., Tan R., Kumar P., Benitz W., Eichenwald E., Cummings J., COMMITTEE ON FETUS AND NEWBORN (2014). Surfactant Replacement Therapy for Preterm and Term Neonates with Respiratory Distress. Pediatrics.

[58] Chen I.L., Chen H.L. (2022). New developments in neonatal respiratory management. Pediatr. Neonatol..

[59] Bianco F., Salomone F., Milesi I., Murgia X., Bonelli S., Pasini E., Dellacà R., Ventura M.L., Pillow J. (2021). Aerosol drug delivery to spontaneously-breathing preterm neonates: Lessons learned. Respir. Res..

[60] Kaluarachchi D.C., Katheria A., Peebles P.J., Guthrie S.O., Kakkilaya V., Dargaville P.A. (2026). Prophylactic surfactant therapy in the era of less invasive surfactant delivery. J. Perinatol..

[61] Zapata H.A., Fort P., Roberts K.D., Kaluarachchi D.C., Guthrie S.O. (2022). Surfactant Administration Through Laryngeal or Supraglottic Airways (SALSA): A Viable Method for Low-Income and Middle-Income Countries. Front. Pediatr..

